# Revealing the gut bacteriome of *Dendroctonus* bark beetles (Curculionidae: Scolytinae): diversity, core members and co-evolutionary patterns

**DOI:** 10.1038/s41598-017-14031-6

**Published:** 2017-10-24

**Authors:** Juan Alfredo Hernández-García, Carlos Iván Briones-Roblero, Flor N. Rivera-Orduña, Gerardo Zúñiga

**Affiliations:** 1 Laboratorio de Variación Biológica y Evolución, Departamento de Zoología, Escuela Nacional de Ciencias Biológicas, Instituto Politécnico Nacional. Prolongación de Carpio y Plan de Ayala s/n. Delegación Miguel Hidalgo, CP11340 México, DF Mexico; 2 Laboratorio de Ecología Microbiana, Departamento de Microbiología, Escuela Nacional de Ciencias Biológicas, Instituto Politécnico Nacional, Prolongación de Carpio y Plan de Ayala s/n. Delegación Miguel Hidalgo, CP11340 Mexico, DF Mexico

## Abstract

*Dendroctonus* bark beetles comprise 20 taxonomically recognized species, which are one of the most destructive pine forest pests in North and Central America, and Eurasia. The aims of this study were to characterize the gut bacterial diversity, to determine the core bacteriome and to explore the ecological association between these bacteria and bark beetles. A total of five bacterial phyla were identified in the gut of 13 *Dendroctonus* species; Proteobacteria was the most abundant, followed by Firmicutes, Fusobacteria, Actinobacteria and Deinococcus-Thermus. The *α*-diversity was low as demonstrated in previous studies and significant differences in *β*-diversity were observed. The core bacteriome was composed of *Enterobacter*, *Pantoea*, *Pseudomonas*, *Rahnella*, *Raoultella*, and *Serratia*. The tanglegram between bacteria and bark beetles suggests that members of bacterial community are acquired from the environment, possibly from the host tree. These findings improve the knowledge about the bacterial community composition, and provide the bases to study the metabolic functions of these bacteria, as well as their interaction with these bark beetles.

## Introduction

The comprehensive analysis of diverse bacteria associated with specific habitats is a prerequisite to improve understanding of both ecological interactions and the functional role that they have with their hosts^1^. Metagenomic studies have provided a deeper knowledge of the evolutionary history of bacterial communities and how these have coevolved with their hosts^2–6^. They have also revealed the different ecological and evolutionary strategies of bacteria, as well as their specific metabolic capacities for successful colonization of complex habitats, such as the insect gut^7^. Furthermore, these investigations have expanded the knowledge about how communities change when they develop in different habitats, and diets^1,8^, geographical locations^9^, host species^10^, and developmental stages^11^.

Bark beetles represent a successful group of Curculionidae due to their diversification and capacity to use a wide variety of plant tissues (bark, outer sapwood, phloem, pith of twigs, small branches or stems, hard seeds, and roots)^12^. The adaptation and diversification of these insects has apparently been linked to shifts from feeding on ancestral conifers to angiosperms^13^, where association with different microorganisms, particularly filamentous fungi, has been vital^14^.

*Dendroctonus* bark beetles comprise 20 taxonomically recognized species, of which 18 are distributed throughout North and Central America and two are native to Europe and Asia^15^. These beetles colonize conifers (*Larix*, *Picea*, *Pinus*, and *Pseudotsugae*) into the Pinaceae family weakened by drought, diseases, mechanical damage or other factors. These insects live in the inner bark of trees, where both larvae and adults feed on phloem and adults breed. They are an important component for renewal and recycling of nutrients in forests; however, when populations experiment large-scale outbreaks, caused by biological and environmental factors, they can kill a substantial number of healthy trees. They are thus considered one of the most destructive pine forest pests^16^.

Previous studies have used culture dependent methods to characterize the bacterial diversity in the gut of some *Dendroctonus* species and to evaluate the functional role of certain bacteria, in relation to digestive processes^17–19^, nitrogen uptake and metabolism^20–22^, plant compound detoxification^23–26^ and protection of the hosts against microbial antagonists^27^. However, it is unknown whether these bacteria are persistent or transient in all *Dendroctonus* species, hindering to make generalizations about their functional role in the gut.

Recently, studies based on next generation sequencing, have been performed to characterize bacterial communities associated with some of these beetles. For example, Adams *et al*.^24^ described bacterial communities associated with *D*. *ponderosae* and their galleries both in *Pinus contorta* and hybrids of *P*. *contorta*-*Pinus banksiana*; likewise, they reported the existence of genes involved in terpene degradation in these bacterial communities. Aylward *et al*.^28^ characterized the microbiota associated with *D*. *ponderosae* and *D*. *frontalis* as well as their galleries. Durand *et al*.^29^ characterized the endo- and ecto-microbiome of *D*. *simplex*, and Mason *et al*.^30^ studied the bacterial community in host galleries of *D*. *valens*. These same methodologies have also been used to evaluate changes in the bacterial community during the natural development or in laboratory rearing conditions in *D*. *micans*, *D*. *punctatus* and *D*. *valens* ^31^, as well as throughout the life cycle of *D*. *rhizophagus* ^32^. Although these previous studies have expanded the knowledge of bacterial communities, so far the core bacteriome of the *Dendroctonus* gut has not been defined, either in terms of its composition and concerning its possible functional role.

Based on mentioned above, we performed a 454 pyrosequencing analysis of the 16S rRNA gene to: a) characterize the gut bacterial diversity in 13 *Dendroctonus* species; b) compare the *β*-diversity among gut bacterial communities of these *Dendroctonus* species and identify the core bacteriome and; c) determine the ecological association between these gut symbionts and bark beetles.

## Results

### Pyrosequencing data

A total of 520 458 reads was obtained from gut samples of the 13 *Dendroctonus* species *D*. *adjunctus*, *D*. *approximatus*, *D*. *brevicomis*, *D*. *frontalis*, *D*. *jeffreyi*, *D*. *mesoamericanus*, *D*. *mexicanus*, *D*. *parallelocollis*, *D*. *ponderosae*, *D*. *pseudotsugae*, *D*. *rhizophagus*, *D*. *valens*, *D*. *vitei* (Table 1). After quality control, 170 577 reads, with a mean of 6 840 reads per sample, remained for subsequent analysis. A total of 1 450 OTUs at 97% of similarity were defined. The lowest number of observed OTUs was 25 in *D*. *pseudotsugae*, whereas the highest was 99 in *D*. *brevicomis*.

**Table 1:** Localities and geographic references of different *Dendroctonus* species analysed.

Country	species (acronym)	Localities	Latitude	Longitude	Altitude (m)	Host
Mexico						
	*D*. *adjunctus* (DADJ)	Jalisco	19° 35′	103° 36′	3,400	*P*. *hartwegii*
	*D*. *approximatus* (DAPP)	Oaxaca	17° 7′	96° 2′	2,000	*P*. *teocote*
	*D*. *frontalis* (DFRO)	Queretaro	21° 08′	99° 37′	2,900	*P*. *patula*
	*D*. *jeffreyi* (DJEF)	Baja California Norte	30° 54′	115° 30′	3,000	*P*. *jeffreyi*
	*D*. *mesoamericanus* (DMES)	Oaxaca	17° 18′	96° 15′	1,619	*P*. *teocote*
	*D*. *mexicanus* (DMEX)	Oaxaca	17° 32′	96° 30′	2,718	*P*. *patula*
	*D*. *parallelocollis* (DPAR)	Jalisco	19° 50.8′	103° 22.6′	1,906	*P*. *hartwegii*
	*D*. *ponderosae* (DPON)	Coahuila	30°33′	108° 37′	2,400	*P*. *strobiformis*
	*D*. *pseudotsugae* (DPSE)	Durango	23° 32′	104° 50′	2,686	*Pseudotsuga menziesii* var. *glauca*
	*D*. *rhizophagus* (DRHI)	Chihuahua	27° 45′	107° 38′	2,400	*P*. *arizonica*
	*D*. *valens* (DVAL)	Morelos	19° 1′	99° 0.0′	1,906	*P*. *leiophylla*
	*D*. *vitei* (DVIT)	Oaxaca	17° 19′	96° 29′	1,900	*P*. *pseudostrobus*
^*^USA						
	*D*. *brevicomis*	Texas	30° 34′	104° 7′	1,409	*P*. *ponderosa*

^*^USA: United State of America.

The 97% of high quality sequences were assigned to some hierarchical level and the remaining 3.0% was unassignable. OTUs were included in five phyla, eight classes, 22 orders, 34 families and 64 genera.

**Figure 1:**
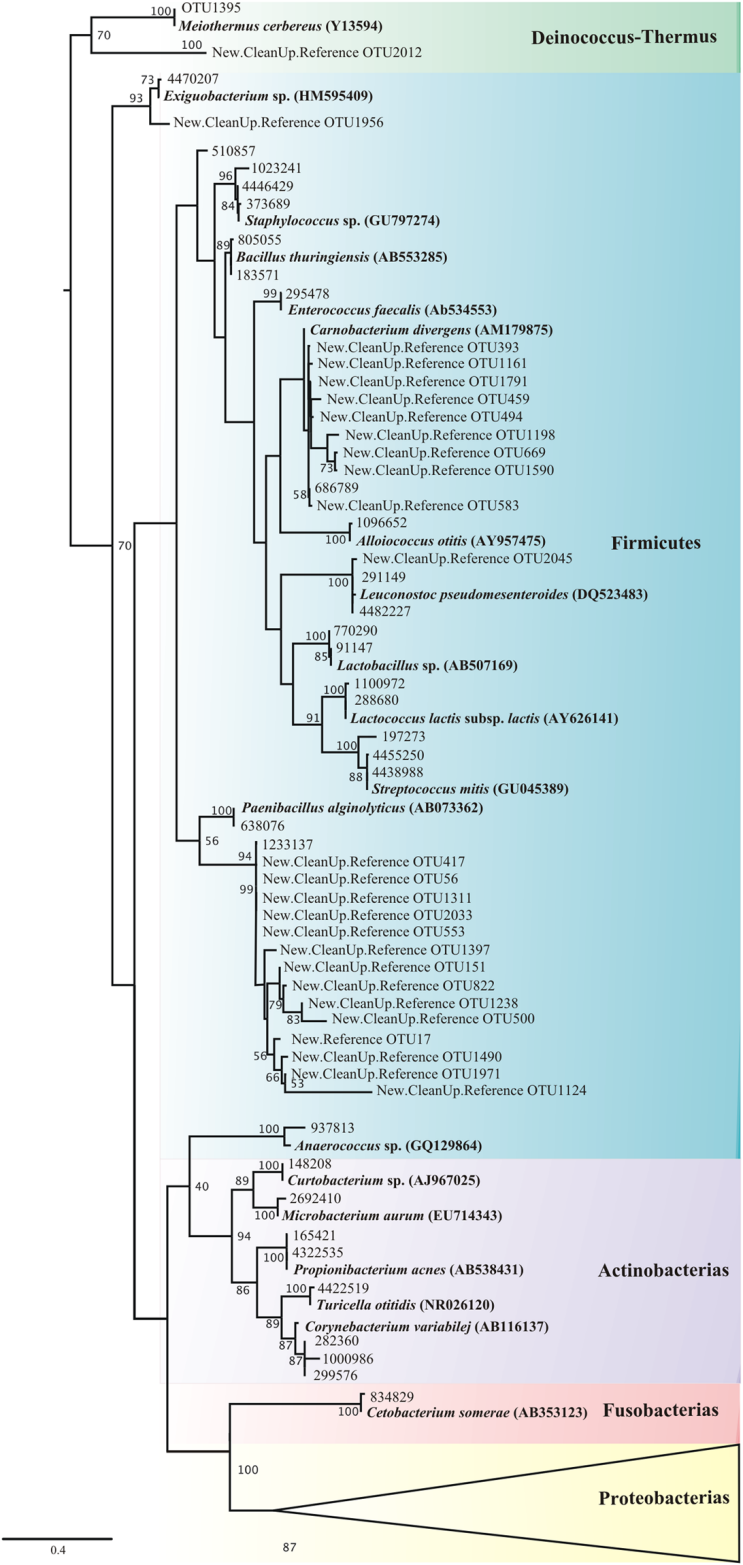
Maximum likelihood phylogeny of the 16S rRNA sequences of the gut bacterial communities of *Dendroctonus* species analysed. Bootstrap values > 0.5 are shown. Phylogeny sections are colour-code according to bacterial phyla and orders, it shows all OTUs found, except those belonging to the Proteobacteria Phylum.

**Figure 2:**
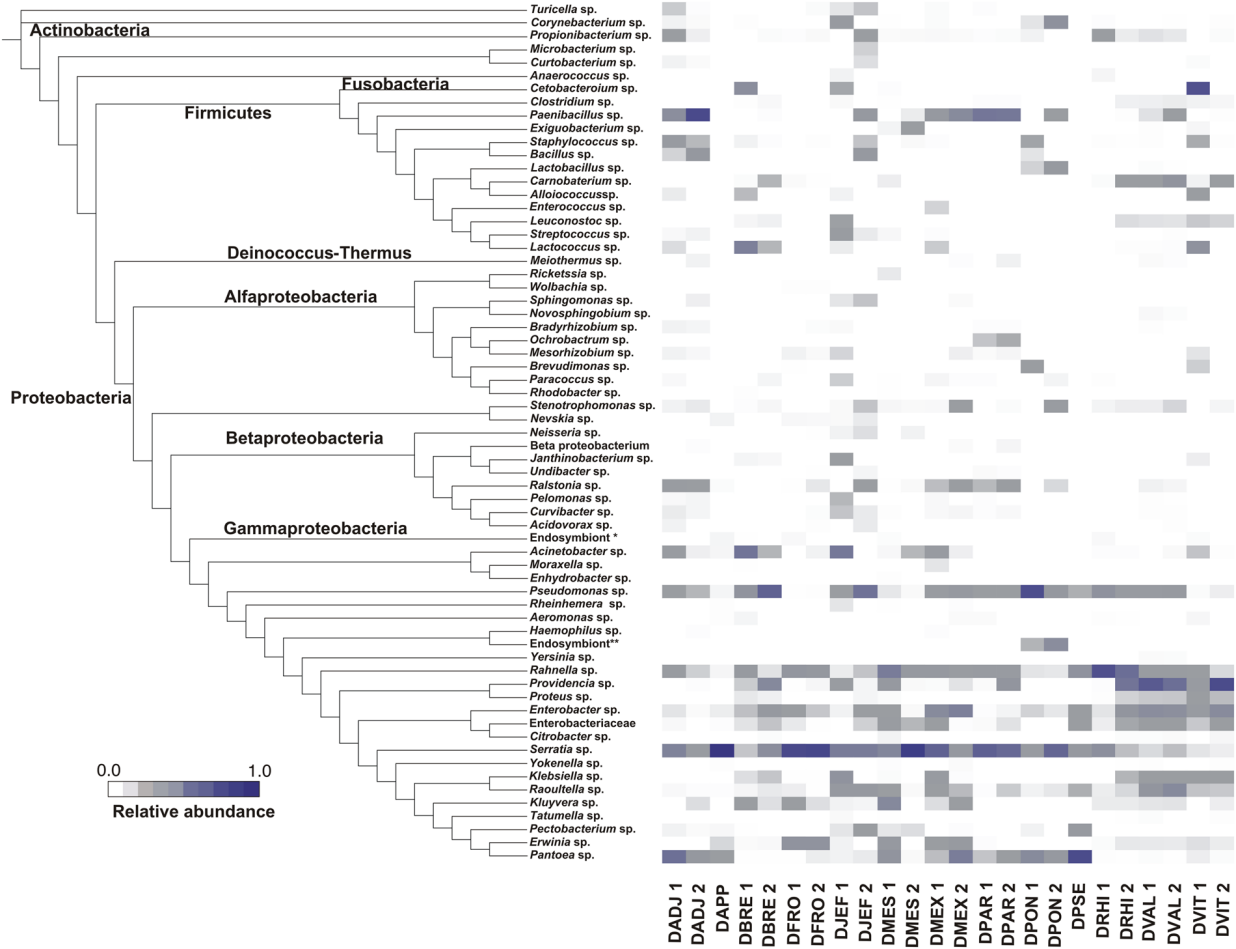
Heat map of representative sequences of operational taxonomic units (OTUs) across different *Dendroctonus* species. The range of colours indicates the OTU relative abundance for each sample; dark colours indicate higher abundance and light ones lower abundance. DADJ: *D*. *adjunctus*; DAPP: *D*. *approximatus*; DBRE: *D*. *brevicomis*; DFRO: *D*. *frontalis*; DJEF: *D*. *jeffreyi*; DMES: *D*. *mesoamericanus*; DMEX: *D*. *mexicanus*; DPAR: *D*. *parallelocollis*; DPON: *D*. *ponderosae*; DPSE: *D*. *pseudotsugae*; DRHI: *D*. *rhizophagus*; DVAL: *D*. *valens*; DVIT: *D*. *vitei*. *Bacterium endosymbiont of *Irenimus aequalis*; **Gammaproteobacterium endosymbiont of *Lamellibrachia Satsuma*.

The most abundant Phylum was Proteobacteria, followed by Firmicutes, Fusobacteria, Actinobacteria and Deinococcus-Thermus (Fig. 1). Within Proteobacteria, Gammaproteobacteria was the best-represented class (Supplementary Fig. S1), and the most abundant genera were *Serratia* (28.03%) and *Providencia* (10.8%). Some genera as *Pseudomonas*, *Rahnella*, *Pantoea*, *Enterobacter*, *Acinetobacter*, *Raoultella*, *Erwinia*, and *Kluyvera* were present in a relative abundance between 1.0–9.2% (Fig. 2). Other genera detected in a relative abundance < 1.0% were *Klebsiella*, *Pectobacterium*, *Proteus*, *Citrobacter*, *Enhydrobacter*, *Haemophilus*, *Moraxella*, *Nevskia*, *Rheinheimera*, and *Tatumella* (Fig. 2). Within this same class, 0.59% of reads were assigned only at family level (Enterobacteriaceae). Betaproteobacteria were mainly represented by *Ralstonia* (0.4%) and other genera covering < 0.2% (*e*.*g*., *Acidovorax*, *Aeromonas*, *Curvibacter*, *Janthinobacterium*) (Fig. 2, Supplementary Fig. S1). Likewise, Alphaproteobacteria was represented by genera whose relative frequency was < 0.4% (*e*.*g*., *Bradyrhizobium*, *Paracoccus*, *Rhodobacter*) (Fig. 2, Supplementary Fig. S1).

The phylum Firmicutes, included 14 genera with relative abundances that varied between 8.1% (*Paenibacillus*) and < 0.2% (*Clostridium*, *Anaerococcus*, *Microbacterium*, and *Exiguobacterium*) (Figs 1 and 2). The phylum Fusobacteria was only represented by *Cetobacter* (3.77%); Actinobacteria contained 5 genera (*Propionibacterium*, *Curtobacterium*, *Turicella*, *Corinebacterium*, and *Micrococcus*), all of them with a relative abundance < 0.1% and; Deinococcus-Thermus represented by *Meiothermus* (0.04%). In addition, reads associated to endosymbionts of *Lamellibraquia* (0.05%) and *Irenimus aequalis* (0.68%) were also identified (Figs 1 and 2).

### Diversity patterns

The Good’s coverage estimator indicated a good sampling completeness from 89 to 98%. Samples of *D*. *brevicomis*, *D*. *jeffreyi*, and *D*. *valens* presented the highest number of observed OTUs with 99, 98, and 87.5, respectively (Supplementary Table 1); whereas *D*. *approximatus*, and *D*. *pseudptsugae* showed the lowest ones, with 30 and 25 OTUs, respectively. The expected richness metrics (Chao1 and ACE) were significantly different among *Dendroctonus* species (*F*_Welch_ test: F_Chao1_ = 123.6, P* < *0.05; F_ACE_ = 478.3, P* < *0.05). Two mean groups for each metrics were obtained with the Dunn test^33^ (P < 0.05) in all comparisons performed. Bacterial communities of same bark beetles species integrated these groups. The first group was composed of those highest richness communities whose values varied from 126 (*D*. *frontalis*) to 305 (*D*. *mesoamericanus*) for Chao1, and from 112 (*D*. *frontalis*) to 295 (*D*. *brevicomis*) with ACE; the second one was composed of communities with lower richness value that varied between 28 (*D*. *pseudotsugae*) to 89 (*D*. *ponderosae*) with Chao1, and 25 (*D*. *pseudotsugae*) to 100 (*D*. *ponderosae*) with ACE (Supplementary Fig. S2).

The Simpson’s reciprocal (S^−1^) index showed the presence from one to nine dominant bacterial genera, with a mean of 5 ± 3. Differences among bacterial communities of bark beetle species was supported by the ANOVA (Welch *F* test: F_(S_^−1^_)_ = 93.679, P* < *0.05) (Supplementary Fig. S2). Two mean groups were integrated with the Dunn test (P < 0.05) in all comparisons performed; the first one composed of bacterial communities with more than three dominant genera (*D*. *valens*, *D*. *brevicomis*, *D*. *jeffreyi*, *D*. *vitei*, *D*. *mexicanus*, *D*. *rhizophagus*, *D*. *ponderosae*, *D*. *adjunctus* and *D*. *parallelocollis*), the second one constituted by those communities with only one or two dominant genera (*D*. *pseudotsugae*, *D*. *frontalis* and *D*. *parallelocollis*) (Supplementary Fig. S2).

The phylogenetic diversity (PD) values were also different among bark beetles (ANOVA *F*_Welch_ test: F_(PD)_ = 1627, P* < *0.05). The Dunn test showed the presence of two mean groups (P < 0.05) in all comparisons performed; the first one including those bacterial communities with PD values from 3.1 to 6.8 (*D*. *jeffreyi*, *D*. *adjuntcus*, *D*. *valens*, *D*. *brevicomis*, *D*. *mexicanus*, *D*. *vitei*, *D*. *ponderosae*, *D*. *mesoamericanus* and *D*. *rhizophagus*), and the second one composed of communities with values from 0.4 to 3.0 (*D*. *frontalis*, *D*. *approximatus* and *D*. *pseudotsugae*) (Supplementary Fig. S2).

**Figure 3:**
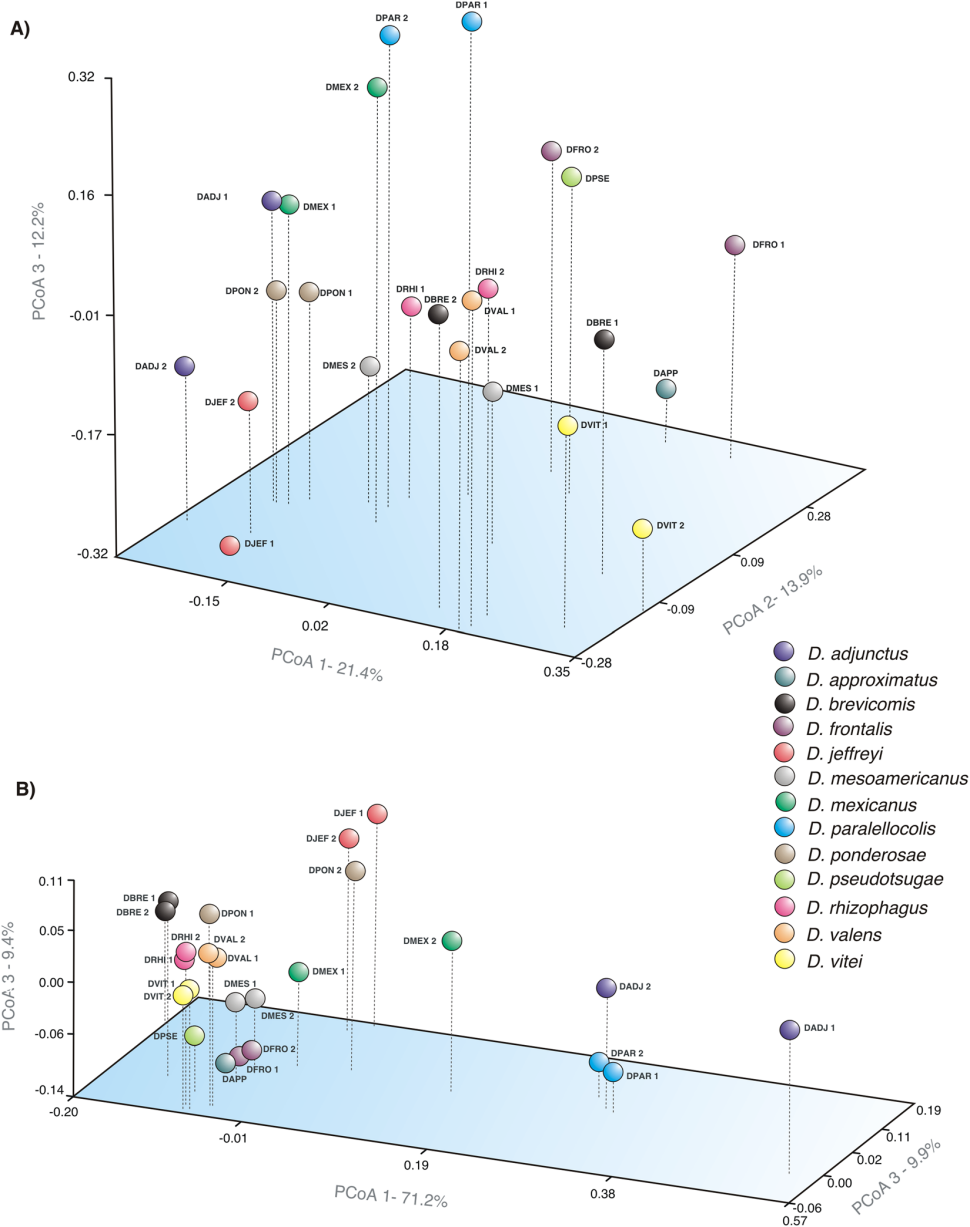
Principal coordinates analysis (PCoA) using unweighted (**A**) and weighted (**B**) UniFrac distances of bacterial communities of *Dendroctonus* species. DADJ, *D*. *adjunctus*; DAPP, *D*. *approximatus*; DBRE, *D*. *brevicomis*; DFRO, *D*. *frontalis*; DJEF, *D*. *jeffreyi*; DMES, *D*. *mesoamericanus*; DMEX, *D*. *mexicanus*; DPAR, *D*. *parallelocollis*; DPON, *D*. *ponderosae*; DPSE, *D*. *pseudotsugae*; DRHI, *D*. *rhizophagus*; DVAL, *D*. *valens*; DVIT, *D*. *vitei*.

The first three principal coordinates of the PCoA using weighted (W) and unweighted (UW) UniFrac distances, explained 90.5% (PCo1-71.2%; PCo2-9.9%; PCo3-9.4%) and 47.5% (PCo1- 21.4%; PCo2-13.9%; PCo3-12.2%) of total variation, respectively (Fig. 3). The PCoA_(UW)_ showed that the bacterial diversity was different (*P* < 0.05) among communities of *Dendroctonus* species, but not between replicates (*P* > 0.05) (*e*.*g*., *D*. *parallelocolis*, *D*. *ponderosae*, *D*. *valens* and *D*. *rhizophagus*) of each insect species (Fig. 3A). On the other hand, the PCoA_(W)_ showed that abundance have a effect on bacterial community, not showing significant differences in the *β*-diversity among bark beetles (*P* < 0.05), except between those associated with *D*. *adjunctus* and *D*. *parallelocollis* (Fig. 3B). Likewise, statistically significant differences were also found among *β-*diversity of *Dendroctonus* species (Adonis test, *P* = 0.001, R^2^ = 0.79) using the Bray-Curtis dissimilarity matrix.

### Core bacteriome

The strict core (100%) of 13 *Dendroctonus* species and their replicates was constituted by six genera (*Enterobacter*, *Pantoea*, *Pseudomonas*, *Rahnella*, *Raoultella* and *Serratia*), whereas relaxed core included eight additional taxa (*Acinetobacter*, *Propionibacterium*, *Providencia*, *Stenotrophomonas*, *Erwinia*, *Kluyvera*, *Paenibacillus*, *Ralstonia* and some unclassified members of the Enterobacteriaceae family). The strict and relaxed core bacteriomes were represented by the 62.6% and 87.8% of total reads, respectively. The incorporation of dominant bacteria previously reported in other *Dendroctonus* species^29,31,34,35^ did not add to our core bacteriome some other genus not found in this study.

**Figure 4:**
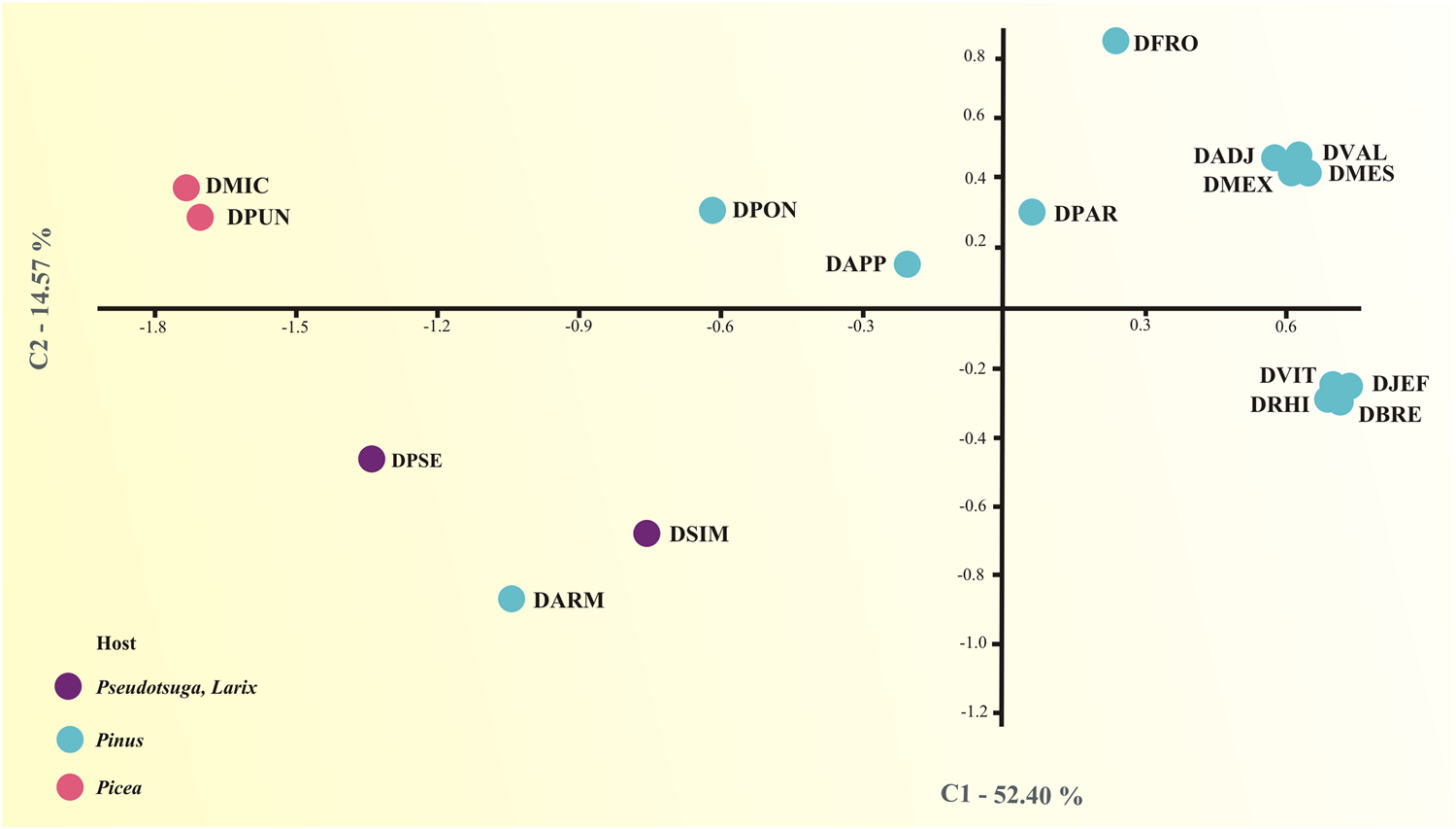
Correspondence Analysis (CA) of the core bacteriome present in the seventeen *Dendroctonus* species. Blue colour indicates the *Pinus* host, purple colour *Pseudotsuga* and *Larix* hosts, and pink colour the *Picea* host. DADJ, *D*. *adjunctus*; DAPP, *D*. *approximatus*; DBRE, *D*. *brevicomis*; DFRO, *D*. *frontalis*; DJEF, *D*. *jeffreyi*; DMES, *D*. *mesoamericanus*; DMEX, *D*. *mexicanus*; DPAR, *D*. *parallelocollis*; DPON, *D*. *ponderosae*; DPSE, *D*. *pseudotsugae*; DRHI, *D*. *rhizophagus*; DVAL, *D*. *valens*; DVIT, *D*. *vitei*.

The two principal axes in the correspondence analysis including the members of the relaxed core explained 63.6% of the total variation (Fig. 4). Three bacterial communities groups were evident in the scatterplot, the first one was integrated bacterial communities of *Dendroctonus* species that feeding exclusively in pines (*D*. *adjunctus*, *D*. *brevicomis*, *D*. *frontalis*, *D*. *jeffreyi*, *D*. *mesoamericanus*, *D*. *mexicanus*, *D*. *parallelocollis*, *D*. *rhizophagus*, *D*. *valens*, and *D*. *vitei*); the second one by bacterial communities of those bark beetles that colonize different species of the *Picea* genera (*D*. *micans* and *D*. *punctatus*); and third one by communities of *Dendroctronus* species that attack *Pseudotsuga* and *Larix* trees (*D*. *pseudotsugae* and *D*. *simplex*, respectively). A special case was bacterial community of *D*. *armandi*, which was separated of other bacterial communities, despite this bark beetle colonizes only one pine species, *Pinus armandi* (Fig. 4).

### Bacterial communities and its association with the bark beetle phylogeny

**Figure 5:**
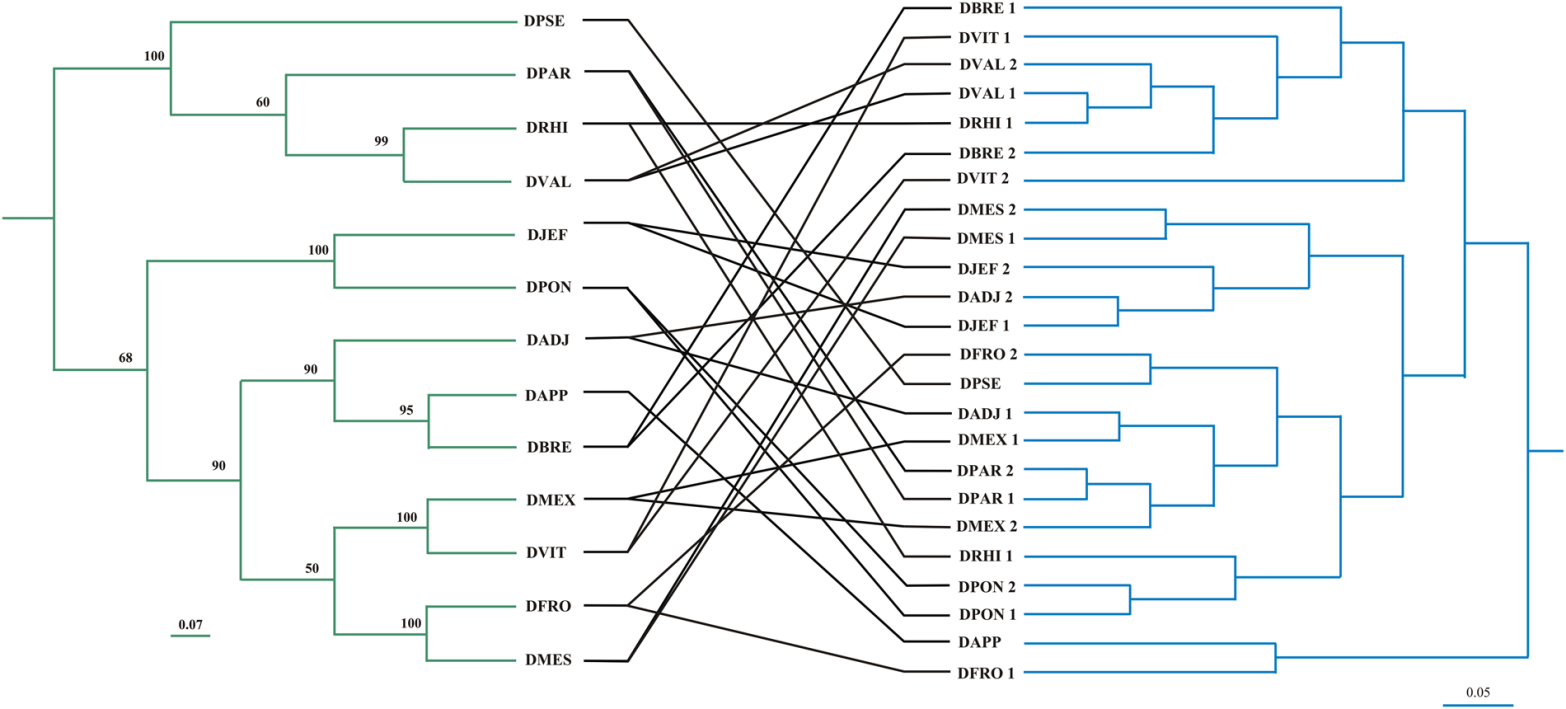
Tanglegram between bark beetles and its bacterial communities (right). Bootstrap values > 0.5 are shown. The scale bar represents 0.07 substitutions per site for the host phylogeny and an unweighted UniFrac distance of 0.05 for the bacterial community dendrogram. DADJ, *D*. *adjunctus*; DAPP, *D*. *approximatus*; DBRE, *D*. *brevicomis*; DFRO, *D*. *frontalis*; DJEF, *D*. *jeffreyi*; DMES, *D*. *mesoamericanus*; DMEX, *D*. *mexicanus*; DPAR, *D*. *parallelocollis*; DPON, *D*. *ponderosae*; DPSE, *D*. *pseudotsugae*; DRHI, *D*. *rhizophagus*; DVAL, *D*. *valens*; DVIT, *D*. *vitei*.

No significant evolutionary congruence between gut bacterial communities and *Dendroctonus* species was found with the tanglegram (Fig. 5). The reconciliation showed that the bacterial microbiota is widespread and the same phylotypes are shared by many species of bark beetles. Three duplication events and host switch, three losses and 5 cospeciation events, were detected for setting 1 (0, 1, 1, 2, 1) and setting 2 (1, 1, 1, 2, 1). In the cases of *D*. *jeffreyi*, *D*. *mesoamericanus*, *D*. *parallelocollis*, *D*. *ponderosae*, and *D*. *valens* a similar gut microbiota was detected in both biological replicates; but this was not the case for the gut bacterial communities of the remaining seven *Dendroctonus* species (Fig. 5).

## Discussion

In this study, we characterized and compared gut bacterial communities from 13 *Dendroctonus* species to identify the core bacteriome, and determined the ecological association between bacterial communities and bark beetles.

Results show the presence of Proteobacteria as dominant taxa, and Firmicutes, Fusobacteria, Actinobacteria, and Deinococcus-Thermus at lower frequencies, which indicate that the structure of *Dendroctonus* gut bacterial communities is similar at the phylum level to those reported in previous studies for other species from this genus^26,28–31^. The detection of 64 bacterial genera across all *Dendroctonus* species, with an average of 24 ± 7.5 taxa per insect species, is similar to detected in other studies in the same genus using this same technology. For example, Durand *et al*.^29^ reported the presence of nine bacterial genera and seven unclassified Proteobacteria and Firmicutes from the surface of cuticle, interior of the body and galleries of *D*. *simplex*; Briones-Roblero *et al*.^32^ recovered 23 bacterial genera analyzing the gut of *D rhizophagus* in all its developmental stages; and Dohet *et al*.^31^ reported 56 bacterial genera in the gut of adults and larvae of *D*. *micans*, *D*. *punctatus* and *D*. *valens* in both field insects and insects grown in the laboratory. In contrast, Xu *et al*.^36^ found 281 bacterial genera in gut and frass samples of both sexes of *D*. *valens* collected in China; the same authors reported 79 bacterial genera in the gut of feeding and non-feeding beetles from this same species, with the monoterpene *α*-pinene^37^.

These differences in the number of bacterial genera reported, particularly in *D*. *valens*, may be associated with the 16S rRNA sequence size, because fragments recovered by means of pyrosequencing are commonly very short (<500 bp). This small size can difficult the taxonomic assignment, especially when an automatic method is used, because it may overestimate the community diversity. The reliability of identification can improve by manually comparing problem sequences with reference sequences deposited in other databases, to corroborate the taxonomic assignment^32,38^, as achieved in this study.

Our results confirm the low *α*-diversity in the gut of *Dendroctonus* species found using the same technologies^29,31,32^_,_ which is comparable to the diversity reported in other insects with a similar diet, such as pine weevils^39^, ambrosia beetles^28^ and some cerambycids^40^. However, this contrast with the high bacterial diversity observed in other herbivorous insects such as ants^41–43^, tree weta insects^44^ and wood-feeding higher and lower termites^38,45^.

The low bacterial diversity observed in bark beetles suggests that several selective pressures within the gut (*e*.*g*., redox potential, pH, microaerophilic conditions, compartmentalization, microbial interactions, and insect immune system) may affect community composition, as has been suggested for other insects^7^. Consequently, similar factors might explain why values of PD and Simpson reciprocal indices are different in the *Dendroctonus* species, despite all of them feed on phloem. In addition, these results suggest that the more aggressive species of *Dendroctonus* bark beetles (*e*.*g*., *D*. *adjunctus*, *D*. *brevicomis*, and *D*. *mexicnaus*) present higher bacterial richness and diversity than those that are not aggressive (*e*.*g*., *D*. *approximatus*, *D*. *parallelocollis*, and *D*. *ponderosae*). However, due to the low replicate number analyzed for each bark beetle in this study, these inferences should be taken with caution.

The heterogeneous *β*-diversity observed in gut bacterial communities of these bark beetles, as it was revealed in the PCoA using unweighted Fast UniFrac (Fig. 3A), is evidently caused by those unshared genera (*e*.*g*., *Klebsiella*, *Paenibacillus*, *Carnobacterium*, *Lactobacillus*, *Acinetobacter*, *Clostridium*, *Tatumella*, *Lactococcus*, *Streptococcus*, *Leuconostoc*, *Cetobacterium*, *Neisseria* and *Alloiococcus*), rather than by members present in core bacteriomes. The effect of these taxa on *β*-diversity is more noticeable when the relative abundance of these taxa is considered (weighted Fast UniFrac), because the PCoA (Fig. 3B) shows that this variation is proportionally less between replicates of the same *Dendroctonus* species than among them, except in the case of *D*. *adjunctus*, *D*. *mexicanus*, and *D*. *ponderosae*.

The factors that cause this variation in *β*-diversity of gut bacterial communities of the same insect species are worthy in further studies, as has been suggested for other insects^46–51^. Given that, the prevalence or not of specific bacteria within the gut is not a random process^7^. In the case of bark beetles, different ecological and demographic factors (*e*.*g*., interactions, competition, population growth, resource availability), as well as microhabitat characteristics mentioned above may determine the presence and/or dominance of bacterial groups and mutualistic relationships between them, at least between members of the relaxed core bacteriome. However, when the physiological conditions of bark beetles change, the pathogenic or commensal capacities of some bacteria may be expressed.

Despite the fact that the microbial community in the gut of insects may vary according to different factors, our results show the presence of a persistent core bacteriome in *Dendroctonus* species. Only members of Enterobacteriaceae and Pseudomonadacea families (*e*.*g*., *Enterobacter*, *Pantoea*, *Pseudomonas*, *Rahnella*, *Raoultella*, and *Serratia*) constitute the strict core bacteriome. The presence of a core bacteriome strongly suggests that the evolutionary history between these bark beetles and their bacterial communities is sustained in the preservation of fundamentals metabolic pathways more than members of this community.

Some members of strict core have been recorded as bark beetle-associated dominant microbes in previous studies using culture-dependent or independent methods^17,21,29–32,34,35,48–51^, indicating that these bacteria may play key roles in different digestive and defensive processes in *Dendroctonus* species. For example, *Pseudomonas* isolates present cellulolytic, lipolytic, esterase, amylolytic and xylanolytic activities^19^, also it has been linked to detoxifying activities in other scolitines^52^, *Rahnella* isolates possess esterase activity and are capable of recycling uric acid^19,22^; some isolates of *Pseudomonas* and *Raoultella* show diazotrophic activity^21,22^; and *Serratia*, *Pseudomonas* and *Rahnella* are able to degrade plant defensive compounds^26^. *Enterobacter* and *Pantoea* have also commonly been isolated from these insects, but their enzymatic capabilities and ecological role have not yet been studied.

Other genera from different families (*e*.*g*., Propionibacteriaceae, Pseudomonadaceae, Paenibacillaceae) are members of the relaxed core bacteriome. Several studies have also reported enzymatic capabilities of these, for example, the genus *Stenotrophomonas* presents cellulolytic activity^17^, *Acinetobacter* shows lipolytic and esterase activities, and *Erwinia* is involved in verbenone production^51^, a compound that acts as anti-aggregation pheromone in these bark beetles.

A noteworthy result derived of the correspondence analysis (Fig. 4) suggests that *Dendroctonus* species colonizing the same host tree (*e*.*g*., *Pinus*, *Picea*, *Pseudotsuga* or *Larix*) share some members (*e*.*g*., *Kluyvera*, *Paenibacillus*, *Acinetobacter* and *Ralstonia*) of the relaxed core bacteriome. The presence of these bacteria in the gut of *Dendroctonus* species that make use of the same host tree, may be explained assuming that these coniferous genera harbor exclusive bacteria; however comparative studies carried out in some *Pinus* species and between *Pinus* and *Picea* reveal that these trees share simple and consistent bacterial communities^53,54^. Another possible explanation is that their presence in the gut is given because they have a complementary ecological role to those displayed by members of strict core bacteriome. Therefore, their retention and persistence might be regulated by both the whole bacterial community itself and by the metabolic contribution that these specific members, together with the strict core bacteriome, bring to these insects. Future studies using metatranscriptomic are necessary to expand our knowledge about the metabolic functions of these gut bacteriome.

Lastly, the tanglegram shows a lack of parallel evolution between bark beetle phylogeny and bacterial communities (Fig. 5), unlike has been observed in other studies realized in termites, cockroaches, corbiculate bees, carrion beetles, fruit flies, and bugs^2,3,5,6,55^.

Several ecological factors including the mechanism of transmission can affect the phylogenetic congruence between bacterial communities and their hosts^56^. Our results suggest that most of gut bacteria of the bark beetles are environmentally acquired, because there is not information about vertical transmission, as it occurs in other insect groups^57,58^. Two hypotheses, which are not necessarily mutually exclusive, are proposed to explain the horizontal transmission of these bacteria in these bark beetles. The first assume that larvae and emerged adults might acquire these microorganisms when they fed on phloem in galleries and colonize new hosts; indeed, a comparison between bacterial communities of the *D*. *rhizophagus* gut and endophytic bacterial (bark, roots and phloem) of healthy saplings of *Pinus arizonica* (one of their preferred pine host) shows that the dominant members of bacteria community (*Rahnella*, *Serratia*, *Pseudomonas*, *Propionibacterium*), in the different life stages of this bark beetle, are present in these pine tissues^32^. The second propose that bacterial communities might be acquired during larval feeding from oral and fecal secretions produced by adults during building of galleries or through the frass yield by themselves during their development.

In summary, all these findings improve the knowledge concerning the gut bacterial composition and diversity of *Dendroctonus* species. These results show the presence of a strict core bacteriome among all analyzed species and show the association of certain bacterial genera (members of the relaxed core) with particular species of these bark beetles. Our results also suggest that bacteria are acquired during emergency, host colonization, and feeding in the larval stage. These results provide a basis for future researches on the functional role that core bacteriome members could have in the gut of these bark beetles.

## Materials and Methods

### Insect collection, dissection and DNA extraction

Emerged adult insects of 13 *Dendroctonus* species were collected directly from infested pine trees in different localities of Mexico and United States between September 2013 and May 2015 (Table 1). In order to integrate two biological replicates for each *Dendroctonus* species, two sets each of 30 insects from five different trees were taken in each locality. The tree bark was removed with chisel and hammer and insects were directly obtained from galleries using fine forceps, placed in sterile plastic containers, stored at 4 °C for their transport to the laboratory and processed immediately once arrived to the laboratory. The identification of *Dendroctonus* species was carried out using a taxonomic key of these insects^59^.

The insects were dissected as described by Briones-Roblero *et al*.^32^. For each biological replicate, three sets of ten guts were processed independently for DNA extraction.

### Bacterial 16S rRNA PCR amplification and pyrosequencing

The V1–V3 region of the bacterial 16S rRNA gene was amplified using the primers 8 F and 556R^60^, fitted to 10 bp multiplex identifiers (MID) and Roche 454 adaptors for Lib-L protocol. PCR reactions were performed in a thermocycler Techne TC 5000 (Staffordshire, UK) on 25-µL of total reaction volume, containing 80 ng DNA template, 1× reaction buffer, 2.0 mM MgCl_2_, 0.4 pM each primer, 0.4 mM each dNTPs, and 1.0 U High Fidelity Platinum Taq DNA polymerase (Invitrogen™ Life Sciences, USA). Reaction conditions involved an initial denaturation step at 94 °C for 5 min, followed by 25 cycles of denaturation at 94 °C for 50 s, annealing temperature at 53 °C for 50 s, an extension at 72 °C for 50 s, and a final extension at 72 °C for 5 min.

PCR products from the three sets of ten guts were mixed and purified using a QIAquick Gel Extraction kit (Qiagen, Valencia, CA). Amplicons for each biological replicate were pooled in equal volumes for pyrosequencing, which was performed using a Roche GS-FLX Titanium 454 pyrosequencer (Roche, Mannheim, Germany) in Macrogen Inc. (Seoul, Korea).

### Data analysis

Pysosequencing data from gut bacteria were analysed using the Quantitative Insight into Microbial Ecology (QIIME) software version 1.8^61^. Because the number of reads was not abundant in the DAPP-2 and DPSE-2 libraries, these species had no biological replicates. Two libraries (biological replicates) of gut microbiota from emerged adults of DRHI (SRP066495)^32^ extracted from the GeneBank database were included in the analysis. Other bark beetle libraries, deposited in this database, were not incorporated in the study, because they analyzed other parts of the insect, rather than just the gut.

Low quality reads with length < 200 or > 500 nucleotides (nt) or containing ambiguous characters, quality score (Phred) < 25, non-exact barcode sequence, presence of homopolymers (>6 nt) or mismatches in primer sequences (>14) were excluded from the subsequent analyses^62^. High-quality reads were grouped in Operational Taxonomic Units (OTUs) using the open-reference method at a similarity threshold of 97%^63^ with Uclust OTUs picker version 1.2.22^64^. Chimeric sequences were detected using Chimera Slayer^65^ and then removed. For each OTU, one representative sequence was extracted (the longest and most abundant) and aligned to the Greengenes set (available from http://greengenes.lbl.gov/) using PyNast program^66^.

The taxonomic assignment for each hierarchical level from phylum to genus was estimated for the representative sequences at 80% of confidence threshold using the Ribosomal Database Project (RDP) Naïve Bayesian Classifier (http//rdp.cme.msu.edu/classifier/classifier.jsp). In order to corroborate the taxonomic assignment of OTUs, we manually compared the acquired sequences with those deposited in three databases: RDP, GenBank (http://blast.ncbi.nlm.nih.gov/Blast.cgi) and Greengenes (http://greengenes.lbl.gov/). A phylogenetic inference analysis was performed using the Maximum likelihood algorithm implemented in PhyML (http://atgc.lirmm.fr/phyml/). Prior to analysis, representative sequences and those of reference sequences downloaded from these databases were aligned in Clustal X v2.0.10^67^, and trimmed at their 5′ and 3′ ends to obtain fragments ≈ 400 bp. A nucleotide substitution model and relevant model parameters were determined for this sequence set in JModeltest v2.1.7^68^ using the Akaike Information Criterion (AIC); the most supported model was General Time Reversible GTR + γ. We selected optimization of tree topology, rather than branch length. The reliability of each node was estimated via a bootstrap analysis after 1000 pseudoreplicates. Likewise, another ML-tree was built using the representative sequences following the method previously described using the GTR + γ model employing the OTUs with highest relative abundance to generate a heatmap in the web page of Interactive Tree of Life, iTol (http://itol.embl.de/).

### Analysis of bacterial communities

We homogenized the numbers of reads with respect to the sample with the lowest counts, in order to characterize gut bacterial communities and to avoid bias in diversity estimation. The Good’s coverage estimator (the probability that a randomly selected amplicon from a sample was previously sequenced) was calculated to determine the extent that sampling was completed^69^, because this is more appropriated than rarefaction analysis for determining sampling coverage. We calculated different estimator’s species richness of bacteria (Chao1and ACE), *α*-diversity (Simpson’s Reciprocal Index) and phylogenetic *α*-diversity (PD) for the gut bacterial community of each *Dendroctonus* species^70,71^.

The normality and homogeneity of variances of these estimators were tested by Shapiro-Wilkinson test and F test^72^. Because diversity indices did not meet the assumptions of equal variances, these were compared among species by mean of ANOVAs coupled with Welch’s F and its respective *post hoc* multiple paired comparisons using Dunn’s test.

The *β*-diversity comparison of gut bacteria among *Dendroctonus* species was performed using Fast UniFrac distances^73^ both unweighted (phylogenetic richness) and weighted (relative abundance and phylogenetic richness), as well as the Bray-Curtis dissimilarity^74^. Significant differences among bacterial communities of *Dendroctonus* species were tested with the Monte Carlo method for Fast UniFrac distances^73^, and Adonis test for Bray-Curtis index.

To explore diversity patterns more complex of bacterial communities of *Dendroctonus* species in a multidimensional space, we performed a Principal Coordinate Analysis (PCoA) using unweighted and weighted Unifrac distances in NTSYS-PC v.2.02j^75^.

### Core bacteriome

The core bacteriome was determinated using QIIME. We selected two cut-offs to define the core bacteriome: 1) OTUs present in 24 *Dendroctonus* samples (100%, strict core), and 2) OTUs present in at least 15 samples (>60%, relaxed core).

A correspondence analysis was performed in PAST ver. 3.14^76^ to explore the ecological association between members of relaxed core bacteriome and *Dendroctonus* species, and indirectly with the insects’ hosts. The presence of core members was coded as binary data (presence-absence). In this analysis, we also included the dominant bacterial genera reported in the gut of *D*. *armandi*, *D*. *micans*, *D*. *punctatus*, and *D*. *simplex* ^29,31,34,35^.

### Gut bacterial communities and their correlation with bark beetle phylogeny

Following Victor and Zúñiga^15^, we recovered the phylogeny of 13 *Dendroctonus* species to determine the association between these bark beetles and gut bacterial communities. We also built a dendrogram by the unweighted arithmetic average clustering method (UPGMA) for the bacterial communities, using β-diversity distances, estimated with the unweighted UniFrac method. The mapping of phylogeny against the community dendrogram was evaluated using the Jane 4.0 program that considers different events: cospeciation, duplication, duplication with host switching, loss, and failure to diverge^77^. We used both edge- and node- based models^55,77^ with the following cost schemes: set 1 parameters: no cost for cospeciation and cost = 1 for all other events; set 2 parameters: cost = 1 for all events. The tanglegram was reconstructed using 1000 generations and a population size of 200. The obtained cost of optimal trees was achieved by randomizing of the microbiota distance tree (beta = −1), or permuting host-microbiota associations after 100 resamplings, respectively.

## Electronic supplementary material

Supplementary Material Figure 1

Supplementary Material Figure 2

Supplementary Material Table 1
