# Supplementary material for: Revealing the gut bacteriome of Dendroctonus bark beetles (Curculionidae: Scolytinae): diversity, core members and co-evolutionary patterns
Source: Sci Rep. 2017 Oct 24;7:13864. doi: 10.1038/s41598-017-14031-6 (PMC5655642; doi:10.1038/s41598-017-14031-6)
Supplement: Supplementary file 1 — Supplementary Material Figure 1 [file 41598_2017_14031_MOESM1_ESM.pdf]

Revealing the gut bacteriome of *Dendroctonus* bark beetles (Curculionidae: Scolytinae): diversity, core members and co-evolutionary patterns.

Juan Alfredo Hernández-García[1], Carlos Iván Briones-Roblero[1], Flor N. Rivera-Orduña*[2], and Gerardo Zúñiga*[1].

[1]Laboratorio de Variación Biológica y Evolución. Departamento de Zoología, Escuela Nacional de Ciencias Biológicas, Instituto Politécnico Nacional. Prolongación de Carpio y Plan de Ayala s/n. Delegación Miguel Hidalgo. CP11340. México D. F.
[2]Laboratorio de Ecología Microbiana. Departamento de Microbiología, Escuela Nacional de Ciencias Biológicas, Instituto Politécnico Nacional. Prolongación de Carpio y Plan de Ayala s/n. Delegación Miguel Hidalgo. CP11340. México D. F.

Short title: Gut bacterial communities of *Dendroctonus*

*Corresponding authors:
Flor Nohemí Rivera-Orduña: email: flor_1413@hotmail.com
Gerardo Zúñiga: email: capotezu@hotmail.com

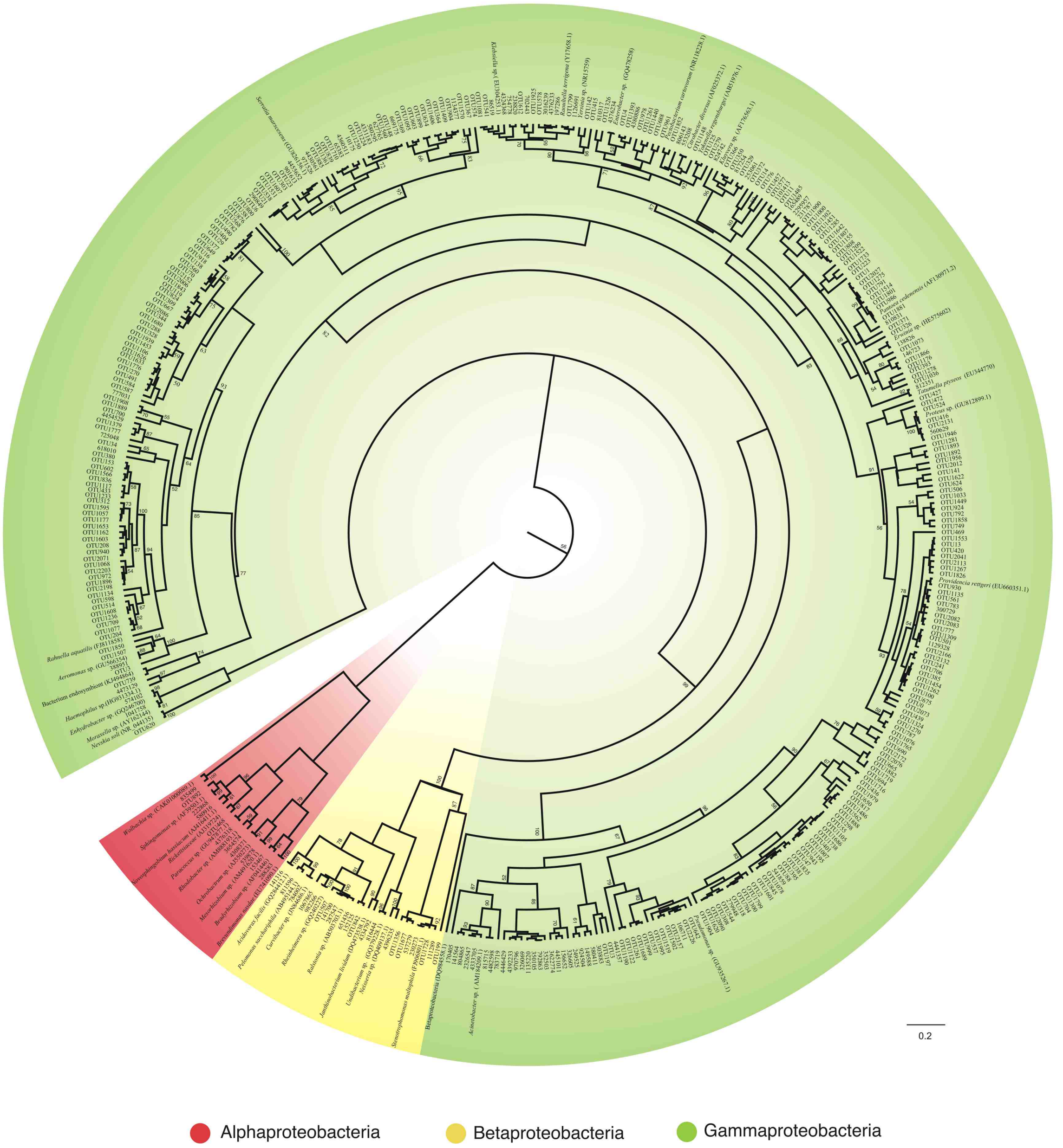

Supplementary material Figure 1. Maximum likelihood phylogeny of the 16S rRNA sequences of the gut bacterial communities of *Dendroctonus* species analysed. Numbers at the nodes are bootstrap values >0.5. Phylogeny sections are colour-code according to bacterial classes; it shows all OTU found belonging to the Proteobacteria Phylum.
