# Supplementary material for: Revealing the gut bacteriome of Dendroctonus bark beetles (Curculionidae: Scolytinae): diversity, core members and co-evolutionary patterns
Source: Sci Rep. 2017 Oct 24;7:13864. doi: 10.1038/s41598-017-14031-6 (PMC5655642; doi:10.1038/s41598-017-14031-6)
Supplement: Supplementary file 2 — Supplementary Material Figure 2 [file 41598_2017_14031_MOESM2_ESM.pdf]

Revealing the gut bacteriome of *Dendroctonus* bark beetles (Curculionidae: Scolytinae): diversity, core members and co-evolutionary patterns.

Juan Alfredo Hernández-García[1], Carlos Iván Briones-Roblero[1], Flor N. Rivera-Orduña*[2], and Gerardo Zúñiga*[1].

[1]Laboratorio de Variación Biológica y Evolución. Departamento de Zoología, Escuela Nacional de Ciencias Biológicas, Instituto Politécnico Nacional. Prolongación de Carpio y Plan de Ayala s/n. Delegación Miguel Hidalgo. CP11340. México D. F.

[2]Laboratorio de Ecología Microbiana. Departamento de Microbiología, Escuela Nacional de Ciencias Biológicas, Instituto Politécnico Nacional. Prolongación de Carpio y Plan de Ayala s/n. Delegación Miguel Hidalgo. CP11340. México D. F.

Short title: Gut bacterial communities of *Dendroctonus*

*Corresponding authors:

Flor Nohemí Rivera-Orduña: email: flor_1413@hotmail.com

Gerardo Zúñiga: email: capotezu@hotmail.com

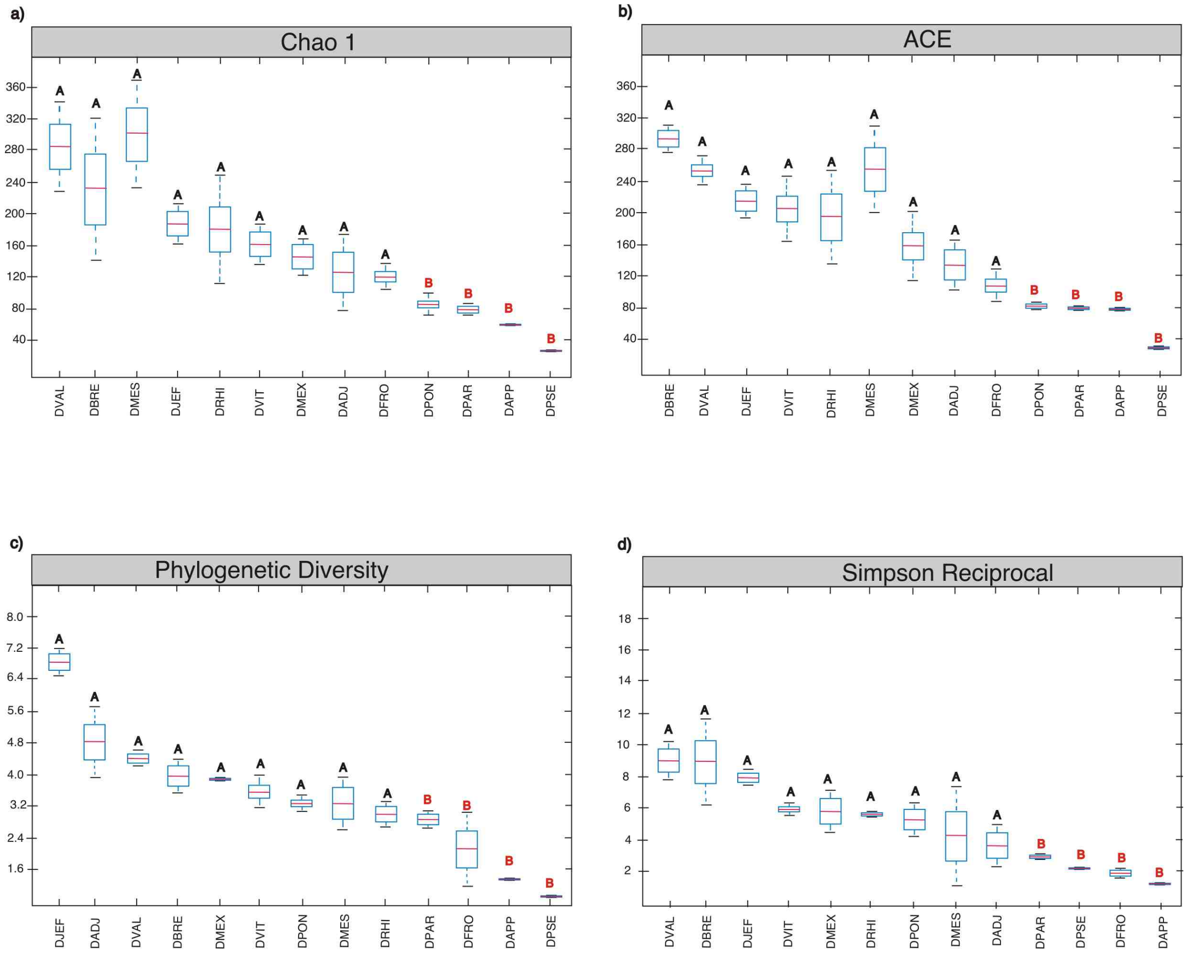

Supplementary material Figure 2. Diversity and richness measurements of the gut bacterial community of different *Dendroctonus* species. Two mean groups are showed to each index, a) and b) richness; c) phylogenetic diversity; and d) Simpsons reciprocal.
