# Supplementary material for: Revealing the gut bacteriome of Dendroctonus bark beetles (Curculionidae: Scolytinae): diversity, core members and co-evolutionary patterns
Source: Sci Rep. 2017 Oct 24;7:13864. doi: 10.1038/s41598-017-14031-6 (PMC5655642; doi:10.1038/s41598-017-14031-6)
Supplement: Supplementary file 3 — Supplementary Material Table 1 [file 41598_2017_14031_MOESM3_ESM.pdf]

Revealing the gut bacteriome of *Dendroctonus* bark beetles (Curculionidae: Scolytinae): diversity, core members and co-evolutionary patterns.

Juan Alfredo Hernández-García[1], Carlos Iván Briones-Roblero[1], Flor N. Rivera-Orduña*[2], and Gerardo Zúñiga*[1].

[1] Laboratorio de Variación Biológica y Evolución. Departamento de Zoología, Escuela Nacional de Ciencias Biológicas, Instituto Politécnico Nacional. Prolongación de Carpio y Plan de Ayala s/n. Delegación Miguel Hidalgo. CP11340. México D. F., México

[2]Laboratorio de Ecología Microbiana. Departamento de Microbiología, Escuela Nacional de Ciencias Biológicas, Instituto Politécnico Nacional. Prolongación de Carpio y Plan de Ayala s/n. Delegación Miguel Hidalgo. CP11340. México, D. F., México.

Short title: Gut bacterial communities of *Dendroctonus*

*Corresponding authors:
Flor Nohemí Rivera-Orduña: email: flor_1413@hotmail.com
Gerardo Zúñiga: email: capotezu@hotmail.com

Table S1. Alpha-diversity estimation of gut bacterial communities of different *Dendroctonus* species.

Label	Observed OTU[97***]	Richness and Diversity index				Good's coverage
		Chao1	ACE****	PD**	Simpson^{-1}	
D. adjunctus	63	131.0	134	4.7	4	0.938
*D. approximatus**	30	68.0	80	1.4	1	0.967
D. brevicomis	99	238.1	295	3.9	9	0.894
D. frontalis	41	126.1	112	2.2	2	0.957
D. mesoamericanus	49	205.6	174	3.3	4	0.950
D. jeffreyi	98	193.3	210	6.8	8	0.908
D. mexicanus	61	151.1	159	3.8	6	0.940
D. parallelocollis	41	82.9	80	3.0	3	0.963
D. ponderosae	46	89.0	100	3.3	5	0.959
*D. pseudotsugae**	25	28.7	25	0.4	2	0.977
D. rhizophagus	57	185.6	192	3.1	6	0.937
D. valens	87.5	293.0	257	4.2	9	0.905
D. vitei	61	167.1	206	3.4	6	0.933

*These samples have not replicates

**Phylogenetic diversity

***Operational Taxonomic Units at 97% of similarity

****Abundance-based in coverage estimator
